# Medically unexplained illness and the diagnosis of hysterical conversion reaction (HCR) in women’s medicine wards of Bangladeshi hospitals: a record review and qualitative study

**DOI:** 10.1186/1472-6874-12-38

**Published:** 2012-10-22

**Authors:** Emily A Kendall, Rashid Uz Zaman, Ruchira Tabassum Naved, Muhammad Waliur Rahman, Mohammad Abdul Kadir, Shaila Arman, Eduardo Azziz-Baumgartner, Emily S Gurley

**Affiliations:** 1 Massachusetts General Hospital, Department of Medicine, Bigelow 740, 55 Fruit St, Boston, MA, 02114, USA; 2 Oxford Policy Management, 6 St. Aldates Courtyard, 38 St. Aldates, Oxford, OX1 1BN, United Kingdom; 3 International Centre for Diarrhoeal Disease Research, Bangladesh (icddr,b), Centre for Equity and Health Systems, 68 Shaheed Tajuddin Ahmed Sarani, Mohakhali, GPO Box-128, Dhaka, 1000, Bangladesh; 4 Center for Communicable Diseases (icddr,b), 68 Shaheed Tajuddin Ahmed Sarani, Mohakhali, GPO Box 128, Dhaka, 1000, Bangladesh; 5 Healthy Communities Research Centre, Building 1, The University of Queensland, 11 Salisbury Road, Ipswich, QLD 4305, Australia; 6 US Centers for Disease Control and Prevention, Epidemiology and Prevention Branch, 1600 Clifton Rd, NE, MS A32, Atlanta, GA, 30075, USA

**Keywords:** Women's health services, Mental health, Somatoform disorders, Conversion disorder, Diagnosis, Physician's practice patterns, Health services needs and demand, Bangladesh

## Abstract

**Background:**

Frequent reporting of cases of hysterical conversion reaction (HCR) among hospitalized female medical patients in Bangladesh’s public hospital system led us to explore the prevalence of “HCR” diagnoses within hospitals and the manner in which physicians identify, manage, and perceive patients whom they diagnose with HCR.

**Methods:**

We reviewed admission records from women’s general medicine wards in two public hospitals to determine how often and at what point during hospitalization patients received diagnoses of HCR. We also interviewed 13 physicians about their practices and perceptions related to HCR.

**Results:**

Of 2520 women admitted to the selected wards in 2008, 6% received diagnoses of HCR. HCR patients had wide-ranging symptoms including respiratory distress, headaches, chest pain, convulsions, and abdominal complaints. Most doctors diagnosed HCR in patients who had any medically-unexplained physical symptom. According to physician reports, women admitted to medical wards for HCR received brief diagnostic evaluations and initial treatment with short-acting tranquilizers or placebo agents. Some were referred to outpatient psychiatric treatment. Physicians reported that repeated admissions for HCR were common. Physicians noted various social factors associated with HCR, and they described failures of the current system to meet psychosocial needs of HCR patients.

**Conclusions:**

In these hospital settings, physicians assign HCR diagnoses frequently and based on vague criteria. We recommend providing education to increase general physicians’ awareness, skill, and comfort level when encountering somatization and other common psychiatric issues. Given limited diagnostic capacity for all patients, we raise concern that when HCR is used as a "wastebasket" diagnosis for unexplained symptoms, patients with treatable medical conditions may go unrecognized. We also advocate introducing non-physician hospital personnel to address psychosocial needs of HCR patients, assist with triage in a system where both medical inpatient beds and psychiatric services are scarce commodities, and help ensure appropriate follow up.

## Background

Unexplained somatic symptoms such as pain, fatigue, and dizziness are common in primary care and general medicine settings worldwide [1,2]. The frequently-changing terminology for such ailments includes “functional”, “psychogenic”, “non-organic”, “somatoform”, “idiopathic”, and “medically-unexplained”, along with the largely-historical terms “hysteria”, “hysterical neurosis”, and “hysterical conversion,” and the now-exclusively-neurological “conversion disorder”. Bodily symptoms without identifiable underlying pathology reveal connections between mental and physical health and are often associated with underlying mood or other psychiatric disorders [3,4] which tend to go under-diagnosed [5].

Symptoms of somatization are more commonly reported by women than men, [6] but they are more strongly associated with emotional distress than with gender [7-9]. Somatization may increase in settings where physical symptoms are more accepted than emotional or psychological symptoms or where treatment for physical illness is more readily available [10]. Indeed, in southern India, higher concern about and sensitivity to stigma has been shown to correlate with patients reporting more somatization symptoms and fewer depressive symptoms [11]. Medically-unexplained physical symptoms pose unique challenges for health care providers. Patients' symptoms are difficult for clinicians to understand; [12] a suspicion that patients are feigning often leads to patient-provider conflict; [12,13] associated health care utilization and costs are high; [14] and treatment, although often possible, requires time and close patient-provider cooperation [13,15].

A 1975 study from Dhaka, Bangladesh, describes frequent psychogenic symptoms among patients, particularly young females, seeking outpatient medical care [16]. In neighboring India, somatic complaints such as body aches, gynecological symptoms, or weakness and tiredness are the principal descriptors used by women with depression, [17] and incidence rates of “hysteria” or “dissociative conversion” from 0.2% to 3.2% were reported in populations field surveys in West Bengal villages [18]. Outbreaks of mass sociogenic illnesses in Bangladesh, including a widely investigated outbreak among adolescent schoolgirls in 2007 characterized by headaches, weakness, and sensory disturbances, [19] further suggest extensive somatization.

In the course of surveillance for hospital-acquired infections in Bangladeshi teaching hospitals, we noted frequent diagnoses of hysterical conversion disorder (HCR) in the admission logs of women’s general medicine wards, prompting us to further investigate HCR within the local medical culture. This article reports the prevalence of HCR diagnoses (as made by their treating clinicians) among adult female patients in two hospitals and describes how physicians identify, manage, and perceive patients whom they diagnose with HCR.

## Methods

We conducted our study at two Bangladeshi government-funded medical college hospitals where investigators had ongoing surveillance, in distinct geographic regions. Both have emergency departments, men’s, women’s, and pediatric general medicine wards, and specialty consultation services including neurology and psychiatry although no psychiatric ward.

For one women’s medicine ward at each study hospital, we reviewed all admission logbooks from the 2008 calendar year to identify patients whose admitting, interim, or discharge diagnoses included HCR. For these case patients, we abstracted age, admission and discharge dates, and initial, interim, and final diagnoses. We performed statistical analyses using Intercooled Stata v9.1.

To better understand how physicians conceptualized HCR and how they diagnosed and treated HCR patients, we conducted key informant interviews at the study hospitals in April-May 2009 with a convenience sample of 13 physicians who regularly cared for HCR patients. We aimed to obtain a sample of physicians of both sexes with diverse seniority and specialization. Semi-structured interviews, conducted in English or through a Bengali-English interpreter, used open-ended questions focused on physicians’ experiences diagnosing and treating HCR patients, and included questions about the criteria they used to make HCR diagnoses, the treatments their patients typically receive, their perceptions about the causes of HCR, and their views of how HCR impacts the hospital system.

Interviews were audio-recorded, transcribed verbatim, and coded manually using codes derived from the original research objectives and from additional emergent themes, ensuring mutually-exclusive and exhaustive coding categories. We analyzed data using content analysis to understand recurrent themes across interviews [20]. Multiple coauthors reviewed the sorted primary data on key themes and reached consensus regarding their valid interpretation. Hospital logbooks provided a source of triangulation regarding the reasons for hospitalization and hospital course of HCR patients. We report illustrative quotations verbatim and otherwise summarize findings.

Institutional review boards at icddr,b and Vanderbilt University approved the study. The Government of Bangladesh and authorities at individual hospitals approved the use of log books. All participants gave written informed consent for participation, and we conducted interviews in private settings. We refer to hospitals as “A” and “B” in the interest of participant confidentiality.

## Results

### Prevalence of HCR diagnosis

**Figure 1:**
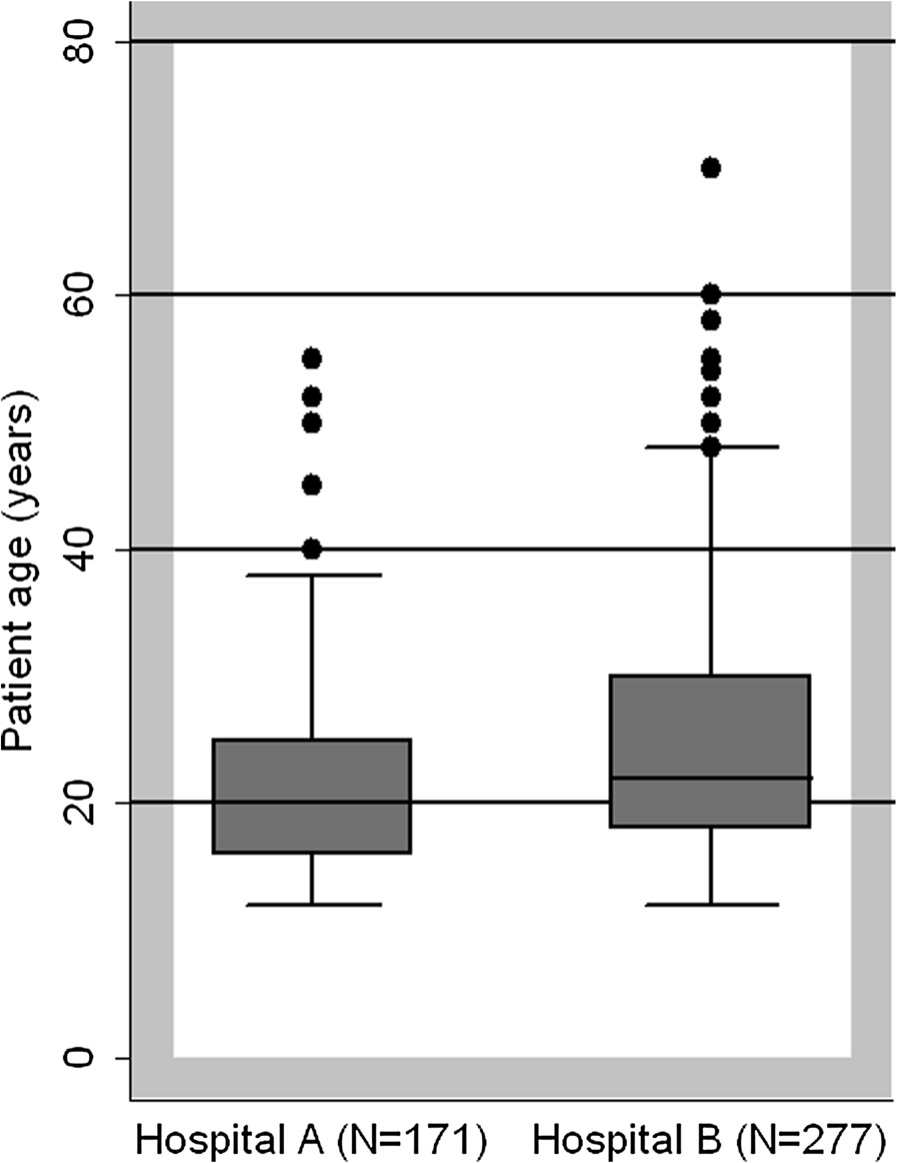
Age distributions of adult female medicine patients diagnosed with hysterical conversion reaction at two study hospitals.

During 2008, Hospital A admitted 2520 patients and Hospital B admitted 5652 patients to the selected women’s medical wards. Among these, 171 (7%) of the 2520 Hospital A patients and 277 (5%) of the 5652 Hospital B patients received HCR diagnoses. Because we were specifically interested in the use of the term "HCR", these tallies exclude diagnoses of “conversion disorder” (8 occurrences), “conversion” (3 occurrences), “functional disease,” and “a case of a psychogenic problem” which also appeared in logbooks, unless the patient also had HCR listed as a diagnosis. The median age of adult female HCR patients was 25 years (interquartile range (IQR) 19–35 years) (Figure 1). The median length of stay for HCR patients in Hospital A (where ~80% of discharge dates were documented) was 2 days (IQR 1–3 days); Hospital B recorded discharge dates too infrequently for analysis.

Sixty percent (267) of the 448 HCR patients retained their HCR diagnoses from admission to discharge (Table 1), and all but 16 of these received the HCR diagnosis exclusively (as opposed to being diagnosed with “HCR and *X”)*. The other 40% of HCR patients had their diagnoses revised to or from a wide range of medical, psychiatric, and syndromic diagnoses (Table 2). A pattern of revision from HCR to some other medical or psychiatric diagnosis or syndrome predominated in Hospital B (where 78 of 100 diagnostic revisions were from HCR to non-HCR), whereas revisions from a non-HCR diagnosis (typically from a physical symptom or syndromic diagnosis) to HCR predominated in Hospital A (where 20 (26%) of 76 diagnostic revisions were from HCR to non-HCR) (Table 1). We also noted twelve cases in which an initial HCR diagnosis was retained while a medical diagnosis such as urinary tract infection, pelvic inflammatory disease, peptic ulcer disease, or hypertension was added, and four cases in which HCR was removed in a revision, leaving only an accompanying medical diagnosis (for example, “HCR with PUD [peptic ulcer disease]” became simply “PUD”).

**Table 1:** Sequential assignment of hysterical conversion reaction (HCR) and alternate diagnoses during case patients’ hospitalizations

**Diagnoses assigned**	**Hospital**		**Total**
	**Hospital A (N= 171)**	**Hospital B (N= 277)**	**(N=448)**
	**Number (%)**	**Number (%)**	**Number (%)**
HCR throughout hospitalization	94 (55%)	173 (62%)	267 (60%)
HCR at admission, changed by discharge	20 (12%)	78 (28%)	98 (22%)
HCR at discharge, not at admission	56 (33%)	22 (8%)	78 (17%)
HCR as interim diagnosis only	1 (1%)	4 (2%)	5 (1%)

**Table 2:** A sampling of diagnoses other than hysterical conversion reaction (HCR) assigned to HCR case patients

Admission diagnoses later revised to HCR*	**No. Patients (N=78)**	Revised discharge diagnoses of patients admitted with HCR diagnosis*	**No. Patients (N=98)**
Symptomatic/Syndromic, not otherwise specified		Medical diagnosis or symptom:	
Respiratory distress or dyspnea	15	Pelvic inflammatory disease	11
Headache	10	Costochondritis	6
Convulsion	9	Urinary tract infection	5
Chest pain	8	Respiratory tract infection	4
Vomiting/diarrhea/abdominal pain	7	Peptic ulcer disease	4
Unconsciousness	4	Sinusitis	3
Fever	3	Enteric fever	3
Restlessness	2	Convulsion	3
Vertigo	2	Bronchial asthma	2
Respiratory distress or dyspnea	15	Migraine	2
Others including palpitation, swelling, weakness, bodyache	1 each	Assorted others (e.g. paroxysmal atrial fibrillation, unstable angina, stroke, pleural effusion, combined deficiency anemia, chronic tonsillitis, electrolyte imbalance, dysmenorrhea, right ovarian cyst, acute calculus, post-menopausal osteoporosis, and fibroadenoma of the breast)	1 each
Psychiatric:			
Generalized anxiety disorder	3		
Anxiety neurosis	2		
Medical:			
Asthma	1		
Ischemic heart disease	1		
		Psychiatric	
		Anxiety neurosis	18
		Generalized anxiety disorder	9
		Acute stress reaction	4
		Panic attack/disorder	3
		Schizophrenia	1

*in some cases multiple non-HCR diagnoses were assigned to the same patient.

### Physician informants

Interviews were conducted with 13 physicians: 5 in Hospital A and 8 in Hospital B. These physicians comprised a range of specialties and seniority levels (Table 3), but few female physicians could be recruited from the predominantly-male hospital staff.

**Table 3:** Characteristics of physicians interviewed about hysterical conversion reaction

**Interview ID**	**Hospital**	**Specialty**	**Seniority level**	**Gender**
1	A	None	Intern	Female
2	A	Internal medicine	Registrar	Male
3	A	Internal medicine	Associate Professor	Male
4	A	Emergency medicine	Emergency medical officer	Male
5	A	Psychiatry	Professor	Male
6	B	Internal medicine	Assistant registrar	Male
7	B	Internal medicine	Indoor medical officer	Female
8	B	Internal medicine	Assistant professor	Male
9	B	Internal medicine	Associate professor	Male
10	B	Emergency medicine	Emergency medical officer	Male
11	B	Emergency medicine	Emergency medical officer	Male
12	B	Psychiatry	Professor	Male
13	B	Neurology	Professor	Male

### Physicians’ definitions of HCR

Physicians characterized HCR as illness with organic-seeming symptoms but without discernible organic etiology. Some physicians considered HCR as a possible diagnosis only when patients presented with neurological syndromes, but most, including the neurologist and psychiatrists, considered HCR when patients presented with any medically-unexplained physical complaint. Convulsions and respiratory distress topped most physicians’ lists of symptoms seen in HCR, and physicians named women in their 20s as the group most likely to have HCR. A few sought a history of personal conflict as a prerequisite to making an HCR diagnosis.

Physicians had learned about HCR by observing more-senior physicians rather than through formal medical school lessons. Some noted that “HCR” was considered outdated terminology outside of Bangladesh, and most of these physicians equated HCR with conversion disorder, dissociative disorder, or hysterical personality disorder as termed elsewhere. The remaining physicians, however, felt that HCR was a problem either quantitatively or qualitatively unique to Bangladesh, and several expressed dismay at the insufficient published literature or academic interest regarding HCR.

### Views about psychological basis of HCR

Physicians considered HCR to occur in reaction to stress and psychological conflict, often as a recurrent and maladaptive response. They named a variety of stressors they felt contributed to HCR. Family and marital disharmony, including upcoming arranged marriages, conflicts with in-laws, and neglect by husbands, were the most commonly cited triggers of HCR. Several senior physicians also specifically cited domestic violence or sexual abuse as potential triggers. Poverty, malnourishment, and financial worries were other commonly-cited stressors. In younger patients, scholastic exam season was observed to trigger an increased frequency of HCR cases, and many physicians also connected HCR with “love affairs,” or dating relationships that ran counter to cultural norms or family marriage arrangements. “A young girl,” one physician described, “about 18 years old, a college student, was in love with a classmate, but her father wouldn’t consent [to the relationship]. He confined her to the house, made sarcastic comments… Gradually the patient developed fits and pseudoseizures. Doctors in the medicine department diagnosed the case as HCR. After two or three weeks [of outpatient psychiatric treatment], she was okay” (5).

Physicians perceived HCR symptoms as a nonverbal declaration of problems by young or uneducated individuals who lacked the communication skills, insight, or support network to express their distress directly. HCR illness could be a way of escaping from unpleasant circumstances or of seeking attention, sympathy, or assistance from husbands or other family members.

Physicians disagreed about whether HCR was distinct from malingering. Many felt that HCR patients had no conscious control over their symptoms. Other physicians, however, suspected HCR patients of deliberately seeking attention or respite; in support of this opinion, they observed that HCR symptoms sometimes appeared and disappeared suddenly depending who was present, and that HCR patients sometimes convulsed or fell without hurting themselves, suggesting willful attention seeking.

### Evaluation of suspected HCR patients

Young, healthy women with normal vital signs brought to the emergency department after acute onset of severe symptoms frequently received provisional diagnoses of HCR. In some cases, emergency physicians assigned these diagnoses within seconds of the patients’ arrival. Some of these HCR patients, especially those with hyperventilation, recovered rapidly and returned home after receiving interventions intended to calm them, such as supplemental face mask oxygen. Typically, however, emergency physicians reported that suspected HCR patients were admitted to the medical ward. Medical reasons for admission included severe symptoms, comorbidities, and diagnostic uncertainty. In addition, demands by patients’ families for further treatment of patients’ complaints, as well as pressure to vacate emergency room beds for new patients, resulted in additional admissions.

On the medical wards, interns evaluated patients and assigned diagnoses upon admission. Interns estimated that senior physicians later amended their HCR diagnoses about 20% of the time. Senior physicians described how their own history-taking delved into the circumstances surrounding the onset of HCR symptoms, and they repeatedly asked patients or their accompanying relatives or friends about any arguments, disappointments, or stressful situations that could have triggered conversion. A few physicians mentioned that they were vigilant for hints of physical or sexual abuse when talking with HCR patients, although none indicated that they routinely asked explicit questions about abuse.

Physical examinations of suspected HCR patients included basic examinations of the heart, lungs, and abdomen, along with symptom-specific assessments such as palpating rib cartilage for tenderness in patients with chest pain and looking for bite marks on the tongues of patients with convulsions. Additional tactics for exploring whether a symptom was psychogenic included observing whether a limp, supine patient whose hand was released over her allowed it to land on her face, watching for corresponding movement of a “paralyzed” leg when the contralateral leg was moved, and monitoring repetitive convulsive movements for inconsistency. Certain HCR management strategies blurred the line between diagnosis and treatment. Physicians described the threat or actual use of nasogastric tubes, urinary catheters, wide-bore needles, or electric shocks – “punishments” (6), in words of one physician – as a routine method of trying to provoke unresponsive patients to respond.

Physicians reported that they used laboratory or imaging studies in only a minority of HCR cases. In particularly-unclear instances, they might order an electrocardiogram, blood cell count, or chest x-ray in a suspected HCR patient with severe or persistent chest pain, or they might refer a patient with severe headaches, vomiting, visual complaints, or convulsions outside the hospital for computerized tomography. Often, however, they did not expect to obtain diagnostic information even from the few investigations they did perform; although four of the physicians mentioned doing blood tests such as complete blood counts and blood glucose levels for certain symptoms in suspected HCR patients, two of the four clarified that these tests were “only to show the patient that we are trying to find out the cause” (8) or so that “the patient thinks that she is under treatment” (11).

When considering HCR, physicians’ differential diagnoses could include asthma, congenital heart disease, depression or anxiety disorders, encephalitis, or stroke. Physicians consistently acknowledged that some patients diagnosed with HCR had unrecognized medical problems, although senior physicians believed that medical diagnoses only rarely escaped their detection. During interviews, physicians cited cardiac arrhythmias and intracranial masses as examples of missed medical diagnoses that became recognized only in subsequent hospitalizations. Professors attributed many initial missed diagnoses to the inexperience of junior doctors. Misdiagnoses also resulted from hasty stereotyping by busy doctors of all experience levels. One physician explained, “A patient with mitral stenosis and palpitations may have some family problems, and may have complications with fast ventricular rate, but whenever a young lady with appropriate background comes up with typical [of HCR] history, sometimes when we are overburdened, we don’t even examine the patient, and sometimes we miss the [medical] diagnosis” (8). Also, one professor who suggested an association of HCR with dyspareunia and pelvic infections asserted that many doctors evaluate gynecologic symptoms incompletely and assign HCR labels hastily to patients who might have gynecologic disease.

### Management of HCR

The majority of physicians reported that within the hospital, they or their medical colleagues often treat HCR patients with “sedatives” or “anxiolytics” such as short-acting benzodiazepines and the combination antipsychotic and tricyclic antidepressant flupentixol/melitracen. For agitated HCR patients, they also might use chlorpromazine or haloperidol. When they discharged patients, some internists provided prescriptions, explaining that “we know that most patients ultimately will not go to the psychiatrist, so we try to replace the role of the psychiatrist” (8). If prescribed, typical regimens included a few months’ course of clonazepam together with a selective serotonic reuptake inhibitor (SSRI) or tricyclic antidepressant and sometimes an antipsychotic such as flupentixol or olanzapine.

Besides these psychoactive medications, physicians named a variety of treatments that they gave to HCR patients as placebos. They described measures such as intravenous saline, supplemental oxygen, vitamins, and painkillers as being medically unnecessary for HCR but given because patients and their families requested and expected them. “This way the patient thinks that she is under treatment… Then, in a very short period, she becomes okay” (11). They reported that even sham treatments such as disconnected oxygen masks or intravenous distilled water “readily cured” (7) some HCR patients. One doctor, pointing to intravenous fluid bags hanging throughout his ward, explained, “intravenous support is psychological support” (6). After HCR patients received medical attention, their symptoms tended to resolve rapidly, and they usually returned home within a day or two.

Many physicians felt that talking with patients was more important than medications for treating HCR and preventing its recurrence. Internists and emergency physicians described how they reassured patients while still acknowledging their suffering. They might tell patients, “Definitely you have pain. The good thing is that you don’t have heart disease” (12), or, “Your disease has been diagnosed, you are getting the proper medications, and you will be fine” (7). Sometimes, medical doctors also mediated patients’ interpersonal conflicts themselves, talking with the patients’ family members to sort out disagreements.

Physicians found it difficult to counsel HCR patients, however. First, busy doctors had little time to talk extensively with hospitalized patients. Second, patients and their families believed that dramatic physical symptoms implied life-threatening medical illnesses, and they distrusted doctors who pronounced them physically well. Third, social stigmatization of psychiatric illness in Bangladesh hampered mental health treatment; physicians shied away from assigning or explaining psychiatric diagnoses, and patients who did receive such diagnoses were reluctant to accept or seek treatment for them them. Finally, scarcity of mental health professionals made appropriate treatment difficult to find.

### Follow-up after discharge

Physicians believed that the care psychiatrists could offer—including education about psychogenic symptoms, assistance resolving interpersonal conflicts, and treatment of comorbidities such as anxiety or depression—would in theory help HCR patients, and if any follow-up care was offered to HCR patients. If an appointment with a psychiatrist. Psychiatry referrals were inconsistent, however; a house officer in Hospital B estimated that one third of HCR patients in his hospital received them. Some physicians refused to refer either because psychiatric diagnoses would upset patients or because the physicians doubted the quality of locally-available psychiatric care. Other physicians provided psychiatry referrals but without explaining to the patients what the “HCR” on their discharge certificates meant. These physicians suspected that few of the patients with referrals attended any psychiatry appointments.

### Physician’s frustrations regarding HCR

Although multiple physicians cited empathy as a critical component of effective interactions with HCR patients, they reported that such patients often provoked negative reactions from doctors. HCR’s prevalence, together with how rarely physicians could identify serious medical illness among HCR patients, led physicians to downplay HCR: “It’s a usual problem, why take it seriously, why do a test, it’s okay,” one quoted his colleagues as saying (6). Interns sometimes called HCR patients “disturbing and annoying” and mocked their complaints “like ‘pain … from head to toe!’” (1). Annoyance at HCR patients was compounded by many doctors’ suspicions that HCR patients manufactured their symptoms. “Sometimes because we are overburdened, we think, ‘Oh, she is malingering; give her a diazepam injection and forget [about her]’” (8).

Physicians also begrudged the time and space that HCR patients consumed. Many expressed frustration at the way HCR patients’ often-melodramatic personalities, together with their initially acute-seeming symptoms, distracted medical staff’s attention from other ill patients. Physicians also felt that HCR patients consumed precious hospital space and physical resources. “We are already loaded with patients… Beds are occupied and there is no space even on the floor. So they are hampering our [ability to take] care of other patients” (9).

For medical doctors with little training or experience in psychiatric care, the need to step outside of familiar biomedical frameworks in order to effectively manage HCR was an additional source of unease. A perceived lack of concern about HCR in the broader medical community further confused and disheartened physicians.

### Physicians’ suggested solutions

Physician informants identified both hospital- and societal-level changes that they believed would help reduce the prevalence of HCR within medical wards. First, physicians consistently wanted to increase capacity to treat patients with psychiatric problems. Some wished for expanded psychiatric specialty services, including more training of psychiatrists or creation of inpatient psychiatric facilities. Others favored increased psychiatric training and support, through continuing education and consult-liaison services, for primary care physicians and internists.

Another desired intervention, which would help potential HCR patients before they reached the hospital, was a network of community- or school-based counselors or social workers to assist with domestic conflict resolution or speak with troubled students. Many physicians also suggested that media campaigns to educate the public about the existence of psychogenic illness would be helpful. Finally, some physicians, considering HCR “a social disorder” (13), felt that elevating the status of women in Bangladeshi society was the most critical prevention measure.

## Discussion

### Meaning and frequency of HCR diagnosis

Six percent of women hospitalized during 2008 in the medical wards we studied received diagnoses of HCR. Therefore, physicians in each study ward made an HCR diagnosis nearly every day that they admitted patients.

Despite this commonness of the HCR diagnosis, we encountered ambiguity among physicians regarding the definition, etiology, and management of HCR. Bangladeshi physicians applied the HCR diagnosis to a wide range of acute neurological and non-neurological complaints for which they saw no underlying medical cause, or in some instances to any acute complaint in a young women with a “hysterical” personality. They also reported difficulty distinguishing involuntary psychogenic symptoms from malingering, and in some cases from organic symptoms. This caused HCR to function as a wastebasket term for medically-unexplained patient presentations.

Bangladeshi physicians were correct to note that the term “HCR”, which is outmoded and imprecise, appears rarely in contemporary medical literature. These physicians' intended meaning, however, places the HCR of Bangladesh within a spectrum of somatoform, non-organic, functional, or medically-unexplained illness that is a worldwide problem. They explained HCR using a psychoanalytic conceptual framework similar to the conversion disorder of psychiatry’s current diagnostic manual, [21] although they did not limit HCR's application to syndromes involving neurological deficits.

We doubt that the 6% of patients receiving HCR diagnoses reflect accurately the prevalence of somatoform illness within our study hospitals. Physicians may have over-diagnosed non-organic illness because they lacked resources to fully medically evaluate patients and because they applied vague HCR terminology overly-broadly. Conversely, HCR diagnoses could under-represent somatoform illness, not only because alternate terminology (e.g., conversion disorder) was used on occasion, but also because of patient and provider discomfort with psychogenic diagnoses. The several final diagnoses of “costochondritis” that replaced “HCR”, for example, suggest that physicians were giving medical labels to symptoms such as chest pain which they suspected as functional and for which they had never found a clear organic cause.

Few data on rates of psychogenic illness among medical inpatients are available for comparison. Most studies either are limited to neurological presentations (with prevalences of conversion disorder reported at much less than 1% among US and UK general hospital and emergency ward patients), [22] or they consider somatization in outpatient settings (with prevalences of somatization disorder of approximately 20% in primary care practices at all wealth and development levels in a 1990s WHO survey) [1]. One Danish survey found a 20% prevalence of DSM-IV somatoform disorders among medical inpatients, but many of the disorders they included, such as hypochondriasis and chronic pain, were not the reasons for admission [2].

### Psychiatric support

Although medical doctors anecdotally find counseling effective as treatment for HCR, and although HCR patients may often have other psychiatric comorbidities, doctors lack the time, space, and training to consistently perform counseling themselves. No psychiatrists, social workers, or other trained support staff are available in the inpatient setting to assist them. After patients leave the hospital, psychiatric follow up is limited by availability, geographic access, and stigma. Recent prevalence estimates for mental health disorders in Bangladesh range from 16% [23] to 28%, [24] and 5-6% of women consider suicide each month, [25] but mental health services are scarce. Bangladesh has 7 psychiatrists per 10 million people, (mostly concentrated near Dhaka), [26] compared with 20 per 10 million in neighboring India [27] and 1200 per 10 million in the United States [28]. Clinical psychologists are even rarer, and psychiatric training for generalists and internists is also minimal [26]. Furthermore, less than 0.5% of total government healthcare spending goes toward mental health – a low figure even compared with other low-income countries, which spend an average of 2% of their healthcare budgets on mental health [29].

The physicians we spoke with observed stresses in HCR patients’ lives ranging from financial or scholastic pressure to domestic conflicts and abuse. The standard medical treatments for HCR, such as sedatives and various minimal-impact interventions chosen to make patients feel like they are being treated, do not address social or psychological factors behind patients’ symptoms. The frequent recurrence of HCR that physicians described highlights the inadequacy of such a system. Psychiatric interventions for somaticizing patients do not have to be resource-intensive, however. An American study of patients with medically-unexplained symptoms showed that single counseling sessions – specifically, intensive short-term dynamic psychotherapy sessions in the emergency department – could reduce repeat visits for similar symptoms [30].

### Medical illness and HCR

Admission logs and physicians' remarks suggest that undiagnosed medical problems also underlie some HCR cases. Most HCR diagnoses were made at the time of admission before full diagnostic evaluations were performed. Although we cannot independently validate the diagnoses made, admission logs show that physicians revised some HCR diagnoses as various medical illnesses were identified. Physicians described additional cases in which explanatory medical diagnoses were made long after discharge.

By assigning HCR diagnoses based on initial impressions—especially in the context of these hospitals’ brief inpatient observation periods, limited availability of diagnostic testing, and overburdened staff—doctors make it easier for themselves to potentially dismiss important medical problems. First, underlying organic causes of patient's presenting symptoms may go unrecognized. Among patients diagnosed with conversion disorder, the fraction with unrecognized underlying neuropathology is estimated at around 4%, [31] and even these imperfect these levels of diagnostic accuracy are attained via thorough specialist evaluations [32] or modern diagnostic tools [33] that were not routinely available to most hospitals in low-income countries such as our study hospitals. Comparable rates of missed medical causes for a broader class of acute somatoform diagnoses, as in our study patients, are unknown. Second, even if a patient does have somatoform illness, this diagnosis may prevent other appropriate medical care. Clinicians' recognition of conversion disorder has been noted to impede the diagnosis of unrelated but coexisting medical conditions [33]. Circumspection is warranted when evaluating a wide array of reported symptoms in patients who seem to fit the hysteria profile.

Labeling patients as “hysterical” seemingly absolves the medical team from further medical work up and patient care. It may also propagate gender disparities in health care when physicians (the vast majority of whom were male in our study hospitals) apply the label mostly to women. Using discord in patients’ personal lives as confirmation of HCR diagnoses is similarly fraught with potential to disguise real medical problems. The presence of emotional stress prior to onset of illness has been shown to be a poor differentiator between conversion and organic illness [34]. Not only are interpersonal conflict and “family problems” universal, domestic violence is widespread in Bangladesh, affecting 60% of women at some time in their lives [35]. Coincidence of physical symptoms with arguments at home is likely to frequently occur by chance.

### Limitations

Our qualitative, hospital-based study is unable to fully characterize the burden, causes, or outcomes of HCR in Bangladesh’s medical system. This study is not intended to determine prevalence of somatoform illness on either a hospital or a population level, and it does not independently verify or refute the diagnoses made by treating physicians. Our qualitative data is limited because of a small number of respondents and only a single round of exploratory interviews, and despite efforts to minimize bias in data collection and interpretion, this study's conclusions reflect the opinions and experience of a few individuals. Because we did not directly evaluate patients, our understanding of the typical features of HCR cases is based on physician recall, which may be biased toward the most memorable cases, and on the minority of HCR cases who had alternate diagnoses listed in logbooks, who may not be representative. Also, the admission logs did not allow us to determine outcomes of case-patients diagnosed with HCR, and apart from reports that many HCR patients later return with the same complaints, the long-term morbidity associated with HCR remains unclear.

## Conclusions

Our study suggests that physicians commonly diagnosed young women presenting with shortness of breath, convulsions, pain, and other somatic complaints with HCR for lack of a more definitive diagnosis. Many likely have somatoform disorders, while others may have other medical and psychiatric disease that remains undiagnosed.

Interventions at the levels of medical education and hospital staffing could alleviate harms of the current system to physicians, HCR patients, and other patients in the ward. To help physicians identify and care for somaticizing patients more effectively and to decrease physicians’ sense of frustration, we advocate regular psychiatric training for internists and general practitioners. In addition to teaching physicians to recognize and treat common problems such as depression, anxiety, and somatization in primary care and hospital settings, training sessions should encourage them to remove hysteria terminology from their arsenal of diagnoses and to defer somatoform disorder diagnoses until after some initial medical workup has excluded reasonable organic etiologies of illness. Although expanded psychiatric specialty services would also be desirable for Bangladesh, basic guidance for non-psychiatrists is a first step that could have broader impact. Close, longitudinal relationships with a physician are associated with reduced somatization, [36] and promotion of such doctor-patient relationships should be an aim when educating general practitioners about common mental health problems.

Second, both HCR patients and other patients in hospitals with a large burden of HCR diagnoses could benefit from the introduction of a single, trained, non-physician counselor or social worker into the department of medicine. This individual could speak with patients believed to have somatoform illness, either in the emergency department (as in Abbass et al. above) or during their stay in the medical ward, providing patients with counseling and ensuring appropriate post-discharge follow-up while freeing doctors and nurses to tend to other duties. The existence of such a program could help to legitimize mental health concerns within the hospital environment, promoting more open discussion and better recognition and management of the medical, psychiatric, and social needs of HCR patients.

## Competing interests

The authors declare that they have no competing interests.

## Authors’ contributions

EAK carried out interviews and hospital record reviews, participated in data analysis, and drafted the manuscript. RUZ assisted in study conception, study design, and data collection. RTN assisted in study design and qualitative data analysis. MWR participated in survey development and other study design. MAK participated in study design and data interpretation. SA participated in data collection. EA participated in study design, data interpretation, and extensive manuscript review. ESG conceived of the study, participated in its design and implemation, and helped to analyze the data and develop the manuscript. All authors read and approved the final manuscript.

## Pre-publication history

The pre-publication history for this paper can be accessed here:

http://www.biomedcentral.com/1472-6874/12/38/prepub
